# Early Creeping Attachment Noted after Mandibular Labial Frenuloplasty

**DOI:** 10.1155/2020/3130894

**Published:** 2020-02-12

**Authors:** P. Stylianou, N. Soldatos, E. K. Edmondson, N. Angelov, R. Weltman

**Affiliations:** ^1^Department of Periodontics and Dental Hygiene, School of Dentistry, University of Texas, Health Science Center at Houston, Houston, TX, USA; ^2^Private Practice, San Antonio, TX, USA

## Abstract

Recession of the mandibular central incisors is frequently associated with high frenum insertion. Often times, this recession is accompanied by a lack of sufficient amount of keratinized tissue and absence of attached gingiva. In this case report, an ASA I patient presented with Cairo Recession Type 2 (RT2) and a minimal amount of keratinized tissue on the mandibular central incisors and underwent frenuloplasty in the anterior mandible with the use of a conventional scalpel technique due to high frenum attachment. The results demonstrated creeping attachment of 1.0 mm as early as 10 days postoperatively resulting in complete root coverage and closure of the interproximal space between teeth #24 and 25. A second soft tissue surgery was avoided due to complete root coverage which remained stable at the 6-month follow-up appointment.

## 1. Introduction

Labial, buccal, and tongue frenal attachments are considered normal anatomical structures of the oral cavity providing stability of the lips, cheeks, and tongue, respectively [1]. The frenum attachment is composed of an orthokeratinized or parakeratinized stratified epithelium, nerve fibers, and elastic and collagen fibers that are derived from the orbicularis oris muscle [2]. Abnormal frenum attachments have been implicated in diastema formation between teeth, especially the maxillary and mandibular central incisors, recession of the gingival margins around teeth with subsequent root exposure, shallow vestibule, difficulty in brushing, loss of teeth alignment, and restricted tongue movement [3]. Moreover, abnormal oral frena have been associated with different syndromes such as Ehlers-Danlos syndrome, infantile hypertrophic pyloric stenosis (IHPS), Ellis-van Creveld syndrome, oral-facial-digital syndrome, and holoprosencephaly (HPE), as well as nonsyndromic disorders [1].

Sewerin in 1971 evaluated in a Danish population the different morphological types of maxillary labial frenum. Eight categories were described: (i) simple frenum, (ii) persistent tectolabial frenum, (iii) simple frenum with appendix, (iv) simple frenum with nodule, (v) double frenum, (vi) frenum with nichum, (vii) bifid frenum, and (viii) frenum with two or more variations at a time [4]. In 1974, Placek et al. introduced a classification system for the labial frenum attachment [5]. Typically, the labial frenum inserts in the mucogingival junction. However, gingival, papillary, or papilla penetrating attachments were described by Placek et al. [5] In addition, as a separate parameter, the “pull syndrome” was described as the frequency of the movement of the interdental papilla resulting from the pull of the frenum. The mucosal attachment was found to be more prominent in upper and lower frena followed by the gingival attachment [5]. The “pull syndrome” was found to be associated with gingival, papillary, and papilla penetrating attachments. The presence of the various types of attachments was not associated with age or sex. One very common finding, associated with abnormal frenum attachment, is the shallow vestibule in the anterior mandible, often accompanied by plaque accumulation, inflammation, gingival recession, and root exposure of the mandibular central incisors, especially where the “pull syndrome” is noted. In addition, these teeth also present with a minimal amount or lack of keratinized tissue and attached gingiva [5].

“Creeping attachment” was first reported in 1964 by Goldman et al. and refers to the coronal migration of the gingival margin after a mucogingival surgery in a coronal direction over portions of a previously denuded root [6]. The coronal migration of the gingival margin does not occur at a constant rate but seemed to be the result of successive episodes of recession and creeping. Biologically, the mechanism could be explained through the functional arrangement and maturation of connective tissue [6].

The purpose of this case report is to demonstrate early creeping attachment after frenuloplasty only, with the use of the conventional scalpel technique.

## 2. Case Description

**Figure 1:**
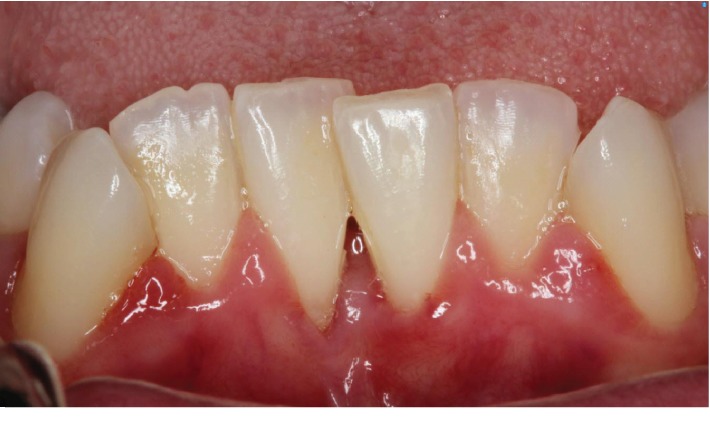
Preoperative picture of tooth #25 presented with 1 mm of recession and 1 mm of keratinized tissue with no attached gingiva. Interproximal loss of papilla and high frenum attachment were noted between #24 and #25.

**Figure 2:**
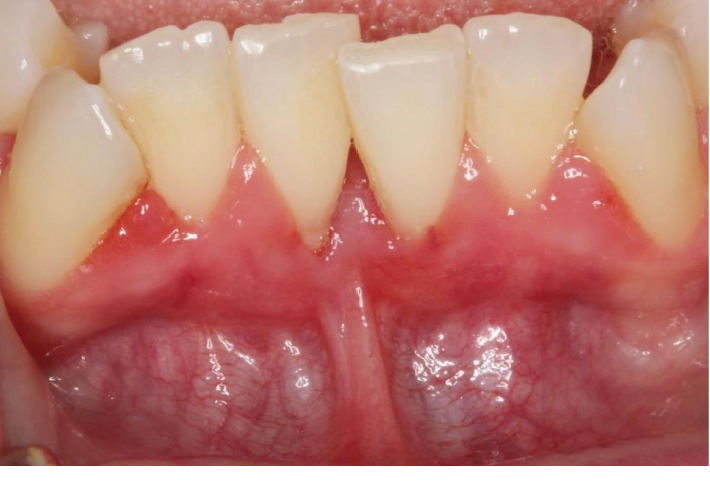
Simple high frenum with gingival attachment was noted between #24 and 25.

**Figure 3:**
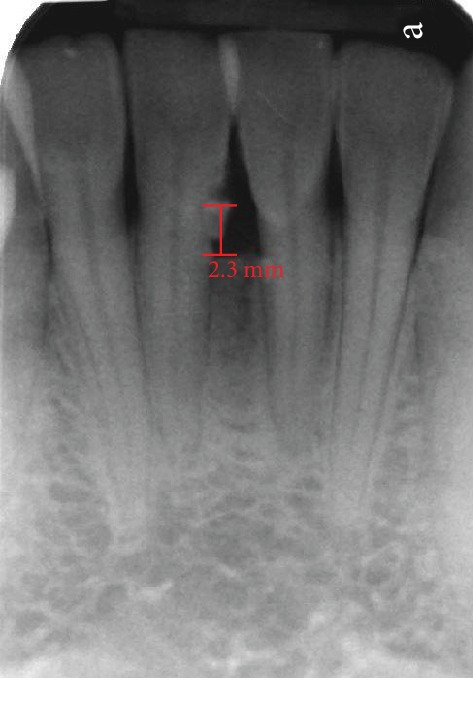
Radiographically 2.3 mm bone loss was noted between #24 and 25.

A 25-year-old ASA I Caucasian female patient presented with a chief concern of sensitivity on the mandibular central incisors. Upon clinical evaluation, teeth #22-27 showed crowding. Tooth #24 presented with no recession and 1.0 mm of keratinized tissue but no attached gingiva, and tooth #25 presented with 1.0 mm of recession and 1.0 mm of keratinized tissue with no attached gingiva. The probing depths and clinical attachment levels on teeth #24 and 25 ranged between 1 and 3 mm. The case was diagnosed with plaque-induced gingivitis on a reduced periodontium and acquired mucogingival deformities around teeth. After the treatment, the patient was placed under regular periodontal maintenance every 6 months. Moreover, on tooth #25, interproximal loss of papilla and a simple high frenum with gingival attachment were noted (Figures 1 and 2). The area between teeth #24 and 25 was diagnosed with “pull syndrome.” Radiographically 2.3 mm of bone loss was noted (Figure 3), and the defect was characterized as Cairo Recession Type 2 (RT2) or Miller class III due to interproximal periodontal attachment loss. Clinically, the probing depths ranged from 1 to 2 mm without BOP. The treatment plan included frenuloplasty, and further assessment for future soft tissue grafting, based on the healing process [7, 8].

**Figure 4:**
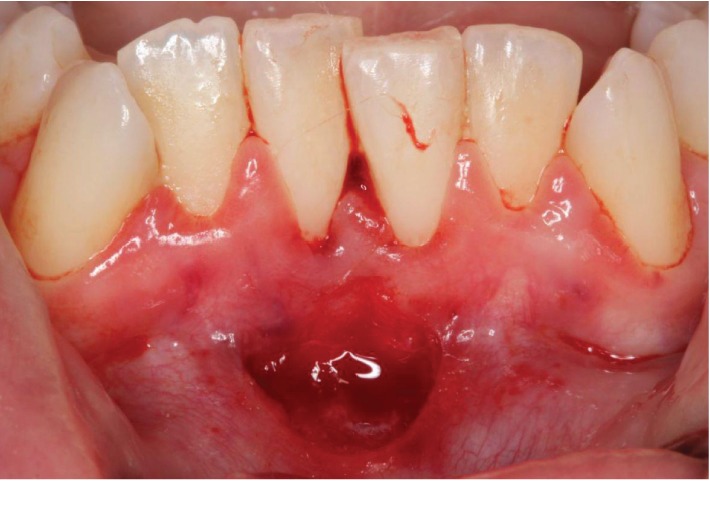
A diamond-shape flap was created using a 15C blade between #24 and 25. The depth of the incision was such to allow for a partial-thickness flap.

**Figure 5:**
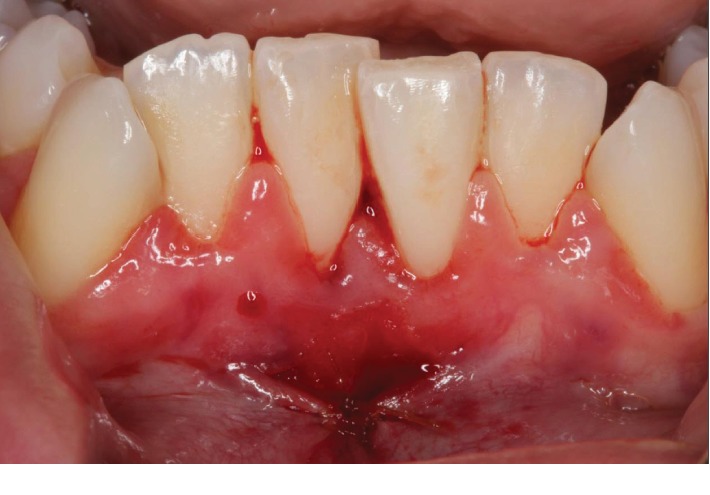
Postoperative picture showed a diamond-shape flap which was secured using 5-0 chromic gut single interrupted suture.

At the day of the surgery, informed consent was obtained for frenuloplasty via a conventional scalpel approach. The area of #24-25 was anesthetized on the labial aspect by means of local infiltrations with one carpule of lidocaine 2% with epinephrine 1 : 100,000. Supragingival scaling was completed on teeth #23-26 for the preparation and smoothing of the root surfaces removing any plaque deposits. A diamond-shape flap was created using a 15C blade. Initially, a “reverse V-shape” incision was made starting from the most coronal aspect of the frenum near its gingival insertion, following the fiber insertion towards the apex, near the vestibule. The depth of the incision was such to allow for a partial-thickness flap (Figure 4). After the initial “reverse V-shape” incision was made, a diamond-shape bed was created, and the vestibular part of the diamond was secured using 5-0 chromic gut single interrupted sutures. With this technique, the vestibular aspect of the diamond flap was left to heal via primary intention and the gingival part near the teeth was left to heal via secondary intention (Figure 5).

**Figure 6:**
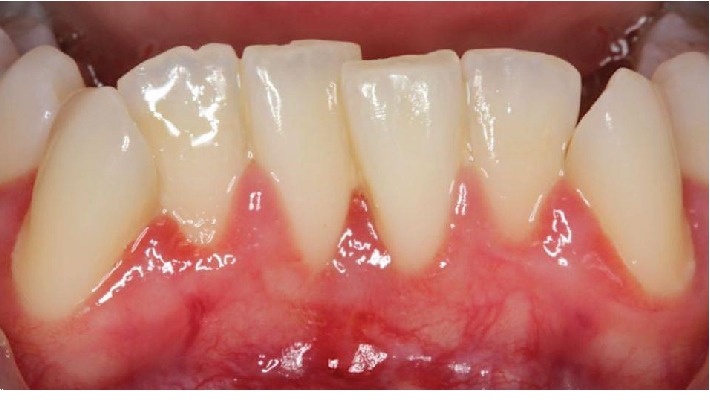
Ten-day postoperative picture showed creeping attachment of 1 mm at #25 and complete closure of the interproximal space, which resulted in complete root coverage.

**Figure 7:**
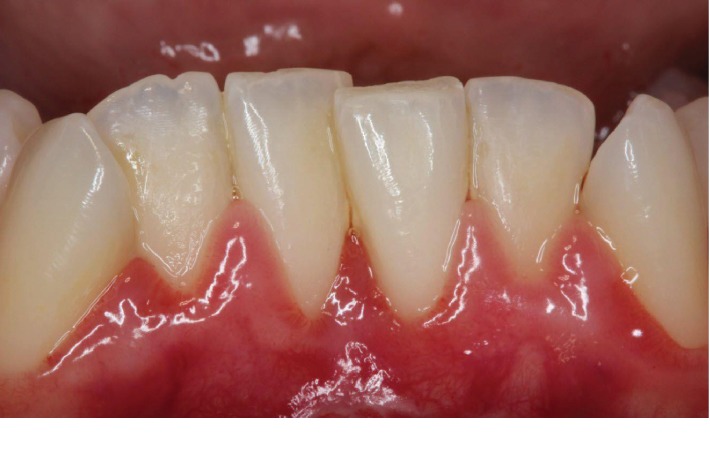
Six-month postoperative picture showed stable clinical results between #24 and 25.

The patient returned at 10 days for a postoperative appointment, and complete root coverage and closure of the interproximal space between #24 and 25 were noted. Additionally, creeping attachment of 1.0 mm was noted on #25, which resulted in elimination of sensitivity issues. The site was healing uneventfully without any signs of infection or inflammation (Figure 6). The patient returned for a follow-up at 1- and 6-month postoperative appointments with stable clinical results (Figure 7). Therefore, a second soft tissue surgery was avoided due to complete root coverage.

## 3. Discussion

It is very important to evaluate the frenal attachments of the patients during routine assessment. Proper diagnosis using the Placek and Sewerin classification systems will result in successful treatment and will prevent severe mucogingival problems like recession and interproximal loss of papilla, teeth malalignment, diastema, and accumulation of plaque due to difficulty in brushing [4, 5].

Labial frenectomy can be performed with a conventional scalpel technique, electrosurgery, or soft tissue lasers [9, 10]. The problem associated with the conventional scalpel technique is the postsurgical pain and discomfort. The increased use of soft tissue lasers in dentistry has also increased patient acceptance [10–12]. Various lasers including CO_2_, diode, Nd:YAG, Er:YSGG, and Er:YAG have been successfully used for the aforementioned procedure [11].

Creeping attachment is frequently observed after mucogingival surgeries, but it does not occur in a constant rate, and the amount is not predictable [6, 13]. Matter in 1980 reported an average of 0.89 mm of creeping attachment 1 year after a free gingival graft surgery [14]. Nelson in 1987 first described creeping attachment of 1-2 mm, during the first postoperative year, in certain cases of subpedicle connective tissue grafting [15]. Similar findings have been reported by Harris, when a connective tissue graft was coupled with partial-thickness double-pedicle flaps, resulting in a mean creeping attachment of 0.8 mm [16]. Most recently, creeping attachment has been reported after a coronally advanced flap and the use of a xenogeneic graft [17]. The creeping attachment reported ranged between 0.5 and 2 mm at 1 year postoperatively [17].

In a similar case report, Fowler and Breault reported a creeping attachment of 1.0 mm after frenectomy with the scalpel technique in the anterior mandible after 4 weeks [3]. In the present case report, the same protocol with Fowler and Breault was followed but the creeping attachment was noted as early as 10 days postoperatively. Furthermore, full coverage of the root surface was noted along with the interproximal soft tissue fill. The results remained stable for 6 months, and a second soft tissue surgery was avoided. Our incidental findings are in accordance with the existing literature and indicate a potential of coronal creeping attachment after removal of high frenal insertions in the anterior mandible which can result in complete root coverage preventing further need for mucogingival surgeries.

## 4. Conclusions

Early creeping attachment of 1.0 mm was noted after removal of high frenum insertion in the anterior mandible which resulted in complete root coverage, preventing further need for mucogingival surgery.
